# The myth of cognitive agency: subpersonal thinking as a cyclically recurring loss of mental autonomy

**DOI:** 10.3389/fpsyg.2013.00931

**Published:** 2013-12-19

**Authors:** Thomas Metzinger

**Affiliations:** ^1^Philosophisches Seminar, Johannes Gutenberg-UniversitätMainz, Germany; ^2^Frankfurt Institute for Advanced StudiesFrankfurt am Main, Germany

**Keywords:** consciousness, self-consciousness, cognitive phenomenology, mental autonomy, subpersonal processes, mind wandering, meditation, epistemic agency

## Abstract

This metatheoretical paper investigates mind wandering from the perspective of philosophy of mind. It has two central claims. The first is that, on a conceptual level, mind wandering can be fruitfully described as a specific form of mental autonomy loss. The second is that, given empirical constraints, most of what we call “conscious thought” is better analyzed as a subpersonal process that more often than not lacks crucial properties traditionally taken to be the hallmark of personal-level cognition - such as mental agency, explicit, consciously experienced goal-directedness, or availability for veto control. I claim that for roughly two thirds of our conscious life-time we do not possess mental autonomy (M-autonomy) in this sense. Empirical data from research on mind wandering and nocturnal dreaming clearly show that phenomenally represented cognitive processing is mostly an automatic, non-agentive process and that personal-level cognition is an exception rather than the rule. This raises an interesting new version of the mind-body problem: How is subpersonal cognition causally related to personal-level thought? More fine-grained phenomenological descriptions for what we called “conscious thought” in the past are needed, as well as a functional decomposition of umbrella terms like “mind wandering” into different target phenomena and a better understanding of the frequent dynamic transitions between spontaneous, task-unrelated thought and meta-awareness. In an attempt to lay some very first conceptual foundations for the now burgeoning field of research on mind wandering, the third section proposes two new criteria for individuating single episodes of mind-wandering, namely, the “self-representational blink” (SRB) and a sudden shift in the phenomenological “unit of identification” (UI). I close by specifying a list of potentially innovative research goals that could serve to establish a stronger connection between mind wandering research and philosophy of mind.

## Introduction: the relevance of mind wandering research for philosophy of mind

Philosophy of mind is not about mental states. It investigates the *concepts* we use to refer to mental states. The philosopher's job is mainly to clarify, differentiate and enrich existing concepts, and sometimes even to develop new conceptual tools to support ongoing research programs. Relatedly, the philosophy of psychology and the philosophy of cognitive science are not about psychological states or cognitive processing *per se*, but about the *theories* we construct about such states and processes, about what counts as an explanation, about what the explananda really are—and about how to integrate different data-sets into a more general theoretical framework. Here, I will offer three simple conceptual tools that may prove helpful: The notion of “mental autonomy (M-autonomy),” the distinction between personal and subpersonal states, and the concept of a “phenomenal self-model” (PSM). At the end I will introduce two new technical terms, the “self-representational blink” and the “unit of identification” (Box 1).

Box 1 **Glossary of Terms.**

**M-autonomy (mental autonomy)**

The term “M-autonomy” (for “mental autonomy” as opposed to autonomy in bodily or social action) refers to the specific ability to control one's own mental functions, like attention, episodic memory, planning, concept formation, rational deliberation, or decision making. This ability can be a form of rational self-control, which is based on reasons, beliefs, and conceptual thought, but it does not have to be. The central defining characteristic is the “veto component”: Being mentally autonomous means that all currently ongoing conscious mental processes can in principle be suspended or terminated. This does not mean that they actually *are* terminated, it just means that the ability exists, and that the person has knowledge of this fact. Functionally, a frequently recurring *loss* of M-autonomy is one major characteristic of our cognitive phenomenology. Phenomenologically, this feature often goes unnoticed.

**V-autonomy (veto-autonomy)**

The term “V-autonomy” (for “veto autonomy”) refers to the ability to voluntarily suspend or inhibit an action. From a logical point of view it is a functional property ascribed not to the brain, but to the person as a whole. This capacity, which could also be called “intentional inhibition,” also applies to mental actions. During a mind wandering episode, we do not have this capacity, because we cannot actively suspend or inhibit our own mental behavior and have no explicit, conscious knowledge of this capacity itself. Conceptually, many forms of mental self-control—like AA—presuppose V-autonomy. Because they are not directly guided by consciously represented reasons, explicit logical inferences, or arguments, however, they are not *rational* forms of self-control.

**AA (attentional agency)**

Paradigmatic autophenomenological report: <**I am a self in the act of controlling my attentional focus**.>

AA typically involves self-identification with an extended body image, plus attentional self-control. It requires a conscious representation of oneself as an entity currently controlling mental resource allocation; and/or having the *potential* for controlling subsymbolic mental resource allocation.

**CA (cognitive agency)**

Paradigmatic autophenomenological report: <**I am a thinking self**.>

CA typically involves self-identification with an extended body image, plus high-level cognitive self-control. It requires a conscious representation of oneself as an entity currently controlling high-level cognitive processing, including quasi-symbolic, conceptual, and propositional contents; and/or having the *potential* for controlling high-level cognitive processing, including quasi-symbolic, conceptual, and propositional content.

**PSM (phenomenal self-model)**

Paradigmatic autophenomenological report: <**I am a self**>

A conscious, global and multimodal representation of the cognitive system as a whole, which may also include psychological and social properties (see Metzinger, 2003a, 2006, 2007 for details).

**EAM (epistemic agent model)**

Paradigmatic autophenomenological report: **<I am a knowing self>**

An EAM is a special type of PSM, allowing a cognitive system to develop a genuine first-person perspective. The EAM refers to a conscious self-representation of the system as an individual entity capable of epistemic self-control, i.e., as currently standing in and/or actively constructing knowledge relations to certain parts of the world; and/or as having the *ability* to actively establish such relations. AA is sufficient for having an EAM, CA is not a necessary condition. The beginning of a mind wandering episode is characterized by a collapse of the EAM.

**SRB (self-representational blink)**

Phenomenologically, the SRB is characterized by a brief loss of self-awareness, followed by an involuntary shift in the UI (see below). Functionally, we can describe it as a failure of attentional and/or cognitive self-control. Alluding to the well-studied phenomenon of the attentional blink (Raymond et al., 1992; Shapiro et al., 1997), the notion of a “self-representational blink” refers to the fact that we are typically not able to consciously experience the actual moment of transition from mindful, present-oriented self-awareness to the identification with the “protagonist” of a daydream, the content of the self-model in autobiographical planning, etc. An empirical prediction of the current account is that subjects should be blind to self-related stimuli during the SRB.

**Personal- and subpersonal-level explanations**

Personal-level explanations are typically *horizontal*, they attribute properties to the person as a whole and proceed from the past to the future in explaining single events by representing a diachronic causal relation as a horizontal line. Subpersonal explanations are typically *vertical*: they concern the relationship between micro- and macro-levels, for example by explaining the abilities or dispositions an organism has in terms of its parts and their causal relations, or by giving a functional analysis that becomes more and more fine-grained. Mind wandering arguably can be explained on the subpersonal level. The question then becomes which forms of conscious cognition, if any, must necessarily be described as personal-level phenomena, which of them are subpersonal processes, and how the relation between personal and subpersonal phenomena can be understood (the “interface problem”).

**Dream/lucidity**

Dreams are complex hallucinatory experience occurring in sleep or during sleep-wake transitions, in which the experiential subject is fully immersed and localized in a spatiotemporal scene. Dreams are characterized by severe, and frequently unnoticed deficits in memory, cognition, and rationality. AA and CA are mostly absent, the EAM is highly unstable (Windt, 2010, 2014; Metzinger, 2013b). “Lucidity” refers to the rare phenomenon that a dreamer is aware of the fact that he or she is currently dreaming. Lucidity can be conceptually analyzed as the gradual stabilization of an EAM and the regaining of M-autonomy. There are different types and stages of lucidity (Windt and Metzinger, 2007; Noreika et al., 2010), and there has been recent progress in isolating the neural correlates of such transitions (Voss et al., 2009, 2013; Dresler et al., 2012). The “meta-awareness” that is regained in dream lucidity may be interestingly related to the termination of mind-wandering episodes during waking periods (Schooler et al., 2011).

**UI (phenomenal unit of identification)**

In most dream and waking states there is a UI, determining the conscious experience of “I *am* this!” In mind wandering and in dreams, the UI can be highly variable, a fact that potentially lends itself to a new way of categorizing individual episodes and thereby constructing a novel taxonomy.

**Minimal UI**

The minimal UI is the *simplest* phenomenal property available for subjective identification. It is not clear what the minimal UI for mind wandering is or how the mechanism of spatiotemporal self-location, a candidate for the minimal UI, works (Windt, 2010; Blanke and Metzinger, 2009).

**Maximal UI**

The maximal UI is the *most general* phenomenal property available for subjective identification. Arguably, this is the experiential quality of the unity consciousness *per se* (i.e., phenomenality *as such*). In some advanced states of meditative practice, during certain kinds of lucid dreams and out-of-body experience the experiential subject seems to be identified with the maximal UI (see Metzinger, 2013b for details).

This paper is composed of three parts. First, I will argue for the claim that one of the most interesting ways of conceptualizing mind wandering is by describing it as a recurring *loss of autonomy*, involving specific kinds of mental self-control. I will then show that for the largest part of our conscious lives we are not mentally autonomous cognitive systems. Part 2 will introduce the distinction between subpersonal and personal events, and argue for the claim that conscious thought should be conceived of as a subpersonal process, with personal-level cognition being the exception rather than the rule: roughly two thirds of conscious human cognitive activity can actually be described as a subpersonal process. Here, the central point is that the transition from subpersonal to personal-level cognition is enabled by a specific form of conscious self-representation, namely, a global model of the cognitive system as an epistemic agent. I argue that the phenomenology of mind wandering can be described as a change in certain functional layers of the PSM. Part 3 will take a closer look at the phenomenology of mind wandering, extracting two novel criteria for individuating episodes of mind wandering and lay the conceptual foundations for future research.

Mind wandering and nocturnal dreaming (cf. Windt and Metzinger, 2007; Fox et al., 2013; Metzinger, 2013a; Wamsley, 2013) are both interesting to philosophers, because both involve sudden shifts in mechanisms of self-identification, rationality deficits, and a cyclically recurring decrease in M-autonomy that is not self-initiated and frequently unnoticed. Generally speaking, autonomy is the capacity for rational self-control, whereas the term M-autonomy refers to the specific ability to control one's own mental functions, like attention, episodic memory, planning, concept formation, rational deliberation, or decision making, etc. Here, my first claim is that the recurring *loss* of M-autonomy is one major characteristic of our cognitive phenomenology^1^ (Bayne and Montague, 2011), and that both research on dreaming and mind wandering have developed important research tools to investigate this hitherto neglected aspect further (like external probing, or systematic questions after sleep lab awakenings; see also Smallwood, 2013; Windt, 2013).

## What is autonomy?

The philosophical concept of “autonomy” has been investigated and refined since antiquity, mostly in the areas of political, legal, and action theory (Pohlmann, 1971; Buss, 2008; Christman, 2008). As it is impossible to even remotely do justice either to the historical literature or to current technical debates, I will, for the purposes of this contribution, first extract four defining criteria from traditional discussions to constitute a semantic precursor for a working concept of “autonomy.” Then, I will narrow down the resulting proto-concept to the domain of mental processes in order to ensure its compatibility with the specific phenomenon of mind wandering. By introducing the notion of “veto autonomy,” or the capacity for intentional inhibition, I suggest a new working concept of M-autonomy which, if successful, can be empirically grounded, gradually refined, and may prove heuristically fruitful in guiding future research.

Autonomy is *rational self-control*. A standard, traditional account of human autonomy would say that autonomy is the ability to control one's own behavior in accordance with reasons and rational arguments. Second, autonomy is *independence* in the formation of one's own will, in the sense of at least potentially having a sufficient degree of independence from one's inner environment (e.g., biases, needs, demands, or past conditioning). It is also the capacity to establish and sustain individual goal-commitments and to impose rules onto one's *own* behavior as opposed to that of others. The idea of “self-governance” captures this semantic element on the social level; according to its historical roots in the political philosophy of Greek antiquity, “autonomy” can also mean opposition to and independence from an outer environment, a tyrant, or the goals, moral commitments, and already existing laws of tradition or one's social group. The third semantic element for autonomy is *self-determination*. This means being able to causally determine your own actions and the decisions that lead to them. The last semantic building block I want to select is *self-formation*: Here, the idea is that you only gradually become a person, a coherent self or rational subject, or even a self-governing agent in the true sense of the word—namely, by becoming more autonomous. Becoming autonomous in this sense is an ongoing process, and it can also be a normative ideal. In sum, rational self-control, a sufficient degree of independence to causally enable individual goal-commitment, “self-governance” and rule-setting, and causal self-determination are all properties that can (and should) be gradually achieved by a human being. They come in degrees, they can always be lost, and they are aspects of a process by which we *become* persons, that is, rational individuals with a coherent, conscious self-representation.

### Mental autonomy

There are not only bodily actions, but also mental actions. Deliberately focusing one's attention on a perceptual object or consciously drawing a logical conclusion are examples of mental actions. Just like physical actions, mental actions possess satisfaction conditions (i.e., they are directed at a goal state). Although they mostly lack overt behavioral correlates, they can be intentionally inhibited, suspended, or terminated, just like bodily actions can. In addition, they are interestingly characterized by their temporally extended phenomenology of ownership, goal-directedness, a subjective sense of effort, and the concomitant conscious experience of global self-control and agency.

Let me distinguish the two most important types of mental action:

- **Attentional agency** (AA), the ability to control one's focus of attention.
- **Cognitive agency** (CA), the ability to control goal/task-related, deliberate thought.

AA and CA are not only functional properties that are gradually acquired in childhood, can be lost in old age or due to brain lesions, and whose incidence, variance, robustness, etc. can be scientifically investigated. They also have a subjective side: AA (Metzinger, 2003a; 6.4.3, 2006, section 4) is also a phenomenal property, as is the case for pain or the subjective quality of “blueness” in a visual color experience (Metzinger, 1995). AA is the conscious experience of actually initiating a shift of attention, of controlling and fixing its focus on a certain aspect of reality. AA involves a sense of effort, and it is the phenomenal signature of our functional ability to actively influence what we will come to know, and what, for now, we will ignore^2^. Consciously experienced AA is theoretically important, because it is probably the earliest and simplest form of experiencing oneself a knowing self, as an epistemic agent. To consciously enjoy AA means that you (the cognitive system as a whole) currently identify with the content of a particular self-representation, an “epistemic agent model” (EAM; Metzinger, 2013a) currently active in your brain. AA is fully transparent^3^: The content of your conscious experience is not one of self-representation or of an ongoing process of self-modeling, of depicting yourself as a causal agent in certain shifts of “zoom factor,” “resolving power,” or “resource allocation,” and so on. Rather, you directly experience *yourself* as, for example, actively selecting a new object for attention. During mind wandering episodes we do not have AA, although these episodes can of course be *about* having been an attentional agent in the past, or *about* planning to control one's attention in the future. Other examples of situations in which this property is selectively missing are non-lucid dreaming and NREM-sleep mentation (Metzinger, 2013a; Windt, 2014), but also infancy, dementia, or severe intoxication syndromes.

An analog point can be made for CA. Conceptually, it is not only a complex set of functional abilities, like the capacity of mental calculation, consciously drawing logical conclusions, engaging in rational, symbolic thought, and so on. There is a distinct phenomenology of currently being a cognitive agent (CA), which can lead to experiential self-reports like “I am a thinking self in the act of grasping a concept,” “I have just actively arrived at a specific conclusion,” etc. What AA and CA have in common is that in both cases, we consciously represent ourselves as epistemic agents. Therefore, the concept of an EAM, refers to a specific type of conscious self-representation. This simply means that on the level of conscious experience, the self is represented as something that either currently stands in an epistemic relation to the world, in the relation of knowing, thinking, actively guiding attention, or actively trying to understand what is going on its environment, or, more abstractly, as an entity that has the *ability* to do so^4^. During full-blown episodes of mind wandering, we are not epistemic agents, neither as controllers of attentional focus nor as deliberate thinkers of thoughts, and we have forgotten about our agentive abilities. A first interim conclusion then is that at the onset of a mind wandering episode, the EAM collapses (see sections The Self-Representational Blink and Mind Wandering as a Switch in the Unit of Identification).

### Losing mental autonomy

Some mental activities are not controllable, because the third defining characteristic does not hold: They cannot be inhibited, suspended, or terminated. Let us call these activities “unintentional mental behaviors.” Mind wandering can now be conceptualized as a form of unintentional behavior, as an involuntary form of mental activity. Of course, the fact that a behavior, be it mental or bodily, is unintentional in no way implies that this behavior is unintelligent or even maladaptive. For example, low-level, saliency driven shifts in attentional focus are unintentional mental behaviors, and not inner actions. In standard situations, they cannot be inhibited. They are initiated by unconscious mechanisms, but may well result in an EAM as their final stage on the symbolic level. Stimulus-independent, task-independent thought is often a form of uncontrolled mental behavior, a breakdown of consciously guided epistemic autoregulation. As long as it is going on, we lack the ability to terminate or suspend it (I will present a reason for this fact in section Making Progress on the Phenomenology of Mind-Wandering below). Yet, from an evolutionary perspective, it may well count as a newly developed, adaptive, and intelligent form of virtual behaviour.

We can now also define a notion of “2nd-order mental action.” The satisfaction conditions of 2nd-order mental actions are constituted by successfully influencing other mental actions or mental behaviors. What Schooler and colleagues have provisionally termed “meta-awareness” (Schooler et al., 2011) is a necessary precondition for 2nd-order mental action. Examples for 2nd-order mental action are the termination of an ongoing violent fantasy, but also the deliberate strengthening and sustaining of a spontaneously arising daydream, the effortful attempt to make an ongoing process of visual perception more precise by selectively controlling the focus of attention, or—in mental calculation or logical thought—the process of imposing a very specific abstract *structure* on a temporal sequence of inner events, of “conducting” a symbolic train of thought (McVay and Kane, 2009). Philosophically, it is interesting to note how 2nd-order mental actions are essential tools for achieving higher degrees of M-autonomy and self-determination; and also how many of them can be described as processes of computational resource allocation in the brain. The potential for M-autonomy and the functional ability to (at least sometimes) operate under a conscious EAM are excellent candidates for criteria of personhood, which have the advantage of empirical grounding and hardware-independence at the same time. It is also important to note that the conceptual distinction between AA and CA either as functional or as phenomenal properties allows for the possibility of *hallucinating* epistemic agency. We might experience ourselves as autonomous mental subjects, but in some cases this might be an adaptive form of self-deception or confabulation (von Hippel and Trivers, 2011). For example, if a subject during an experimental design involving mindfulness-based stress reduction regains meta-awareness (Hölzel et al., 2011; Mrazek et al., 2012) and describes the experience as “I have just realized that I was daydreaming and redirected my attention to the current moment and the physical sensations caused by the process of breathing,” it may be false to assume that, functionally, the “realization” was actually a form of AA or CA (see Schooler et al., 2011, and section The re-appearance of meta-awareness). What is subjectively described or experienced as 2nd-order mental action may sometimes not be a personal-level event at all, but a shift in the subpersonal self-model that is then misdescribed on the level of self-report. In some cases, it may simply be an autophenomenological *post-hoc*-confabulation.

“Veto autonomy” (V-autonomy) is the capacity to voluntarily suspend or inhibit an action, and from a logical point of view it is a functional property which we do not ascribe to the brain, but to the person as a whole. Let us call the capacity in question “intentional inhibition”^5^. During a mind wandering episode, we do not have this capacity, because we cannot actively suspend or inhibit our own mental behavior without thereby terminating the mind wandering episode itself (or by turning it into something else, such as a controlled fantasy, as indicated above, cf. footnote 4). Recent empirical work reveals the dorsal fronto-median cortex (dFMC) as a candidate region for the physical realization of this very special form of purely mental 2nd-order action^6^. It does not overlap with known networks for external inhibition, and its computational function may lie in predicting the social and more long-term individual consequences of a currently unfolding action, that is, in representing the action's socially and temporally more distant implications for the organism^7^. There is a considerable amount of valuable neurobiological data on the physical substrates of intentional inhibition in human beings, and a number of them have already led to more abstract computational models of volitional control, action selection, and intention inhibition itself Brass and Haggard, 2007; Campbell-Meiklejohn et al., 2008; Kühn et al., 2009; Filevich et al., 2012, 2013. These data are valuable not only for understanding the “back end” of many mind wandering episodes (section The re-appearance of meta-awareness), but also for a more comprehensive theory of M-autonomy.

Conceptually, many forms of mental self-control—like AA—presuppose V-autonomy, but are not directly guided by consciously represented reasons, explicit logical inferences, or arguments. Indeed, there is no need or even conceptual necessity to specify autonomy as *rational* self-control, because our capacity for rational self-control is only a special case of a more comprehensive, fundamental set of functional properties. First, rationality does not have to express itself in terms of explicit, symbolic reasoning processes using propositional data-formats (e.g., a Fodorian “language of thought”), but can be operationally defined as a property of some global input-output-function maximizing a specific fitness criterion. Second, there are more operational and empirically grounded models of autonomy, combining the notion of causal self-determination with independence from alternative causes, both inner and outer (see Seth, 2010 for the notion of “G-autonomy” based on a formal analysis of Granger causality). For empirical research programs on mind wandering, such operational concepts are more likely to yield specific, testable hypotheses. Nevertheless, the notion of “rational mental self-control” in the traditional sense remains important if we want to understand the *phenomenology* of high-level cognition and the normative components of our concept of “personhood.” Explicit rational self-control on the mental level cannot be reduced to veto control—on the contrary, the capacity for veto autonomy is one of its centrally relevant constitutive conditions. Clearly, the capacity for inhibiting mental processes via 2nd-order acts of vetoing without the involvement of quasi-conceptual or quasi-propositional representations is the more frequent and also more basic phenomenon, and hence also the more fundamental target for research. You can only be rational if you have the capacity for mental veto autonomy, but you can achieve a high degree of M-autonomy without rational self-control.

### M-autonomy

We can now define a working concept of M-autonomy as the ability to control the conscious contents of one's mind in a goal-directed way, by means of attentional or CA. This ability can be a form of rational self-control, which is based on reasons, beliefs, and conceptual thought, but it does not have to be. What is crucial is the “veto component”: Being mentally autonomous means that all currently ongoing processes can in principle be suspended or terminated. This does not mean that they actually *are* terminated, it just means that the ability, the functional potential, is given and that the person has knowledge of this fact. M-autonomy is the capacity for causal self-determination on the mental level. It has a specific phenomenological profile (see section The Re-appearance of Meta-Awareness), and it certainly comes in degrees, but it is a rather rare property possessed by human beings for only about one third of their conscious life-time. M-autonomy is absent during episodes of mind wandering.

Two points are important in order to avoid potential misunderstandings. First, process and content must not be confused: Mind wandering episodes can certainly be *about* M-autonomy, they can represent past events in which we actually had M-autonomy, or involve the planning of future actions which presuppose M-autonomy, and so on. But because the onset of such episodes will be beyond conscious control, the episode itself is a subpersonal process (see section The Distinction between Subpersonal and Personal Processes). Second, there are interesting situations where human beings quickly alternate between mind wandering and short episodes of M-autonomy. For example, a student may have dissociated from a boring lecture into a pleasant fantasy in which he now indulges. However, from time to time he may “nudge” the fantasy in a certain direction, trying to optimize the daydream, only to deliberately let go again and enjoy the currently pleasant loss of autonomy. The fantasy as a whole, then, is neither an episode of mind-wandering nor a controlled chain of mental actions, but a complex, hybrid process. One may speculate that such hybrid and functionally graded situations are rather frequent (Schad et al., 2012), as in ecologically valid situations, we often seem to move in and out M-autonomy. There is an important conceptual issue here: If we individuate mental episodes by their introspectively accessed content (a specific emotional content or memory element in depressive rumination, a single future task, or an expected situation in autobiographical planning, etc.), then extended chains of mind wandering interspersed with brief, lucid moments of M-autonomy will appear as a single and unified explanandum. A fine-grained functional analysis, however, will likely reveal a large number of individual state transitions (see section The Self-Representational Blink), perhaps also a continuous cyclical path through a state-space the dimensions of which empirical research on mind wandering is just beginning to explore. M-autonomy may certainly be a normative ideal from a wider philosophical perspective, but empirical facts clearly show that we only rarely pass through those regions of our mental state-space to which the concept points^8^.

What about the fourth defining element, the notion of *self-formation*? Regaining M-autonomy—a functional transition that in healthy people probably takes place many hundred times every day—is also self-constitution, because a new type of conscious self-model is created, an EAM, which may change global properties of the system as a whole (e.g., turning it into a subject of experience or being recognized as a rational individual by other cognitive systems). You become more coherent and you construct your own, subjectively experienced transtemporal identity by successfully exerting mental self-control. You do this by constructing an autobiographical self-model from memories of having had M-autonomy, of having successfully controlled, and thereby appropriated, ongoing cognitive activity in the past, successfully making it, phenomenologically, your *own* activity (Metzinger, 2003a, 2007; see section Making Progress on the Phenomenology of Mind-Wandering below). Put simply, under standard conditions what you own is what you can in principle causally control, and this principle holds on the level of mental self-representation as well. Of course, the phenomenal experience of ownership can be manipulated merely by passive exposure to correlations among self-related signals, as in the rubber hand illusion or experimentally induced full-body illusions (Lenggenhager et al., 2007). But agency is more than ownership, it is related to the experience of successful control, and it is also exactly what is relevant in describing the relationship between mind wandering and M-autonomy in terms of the existence or non-existence of an EAM. You can own the thoughts generated by a wandering mind without an EAM (phenomenologically they are still yours) even if the knowledge that you have the causal capacity for self-control is not consciously available, not represented on the level of your PSM. Importantly, this also suggests that rationalizing an earlier episode as having *been* under one's control may be a functionally necessary way of re-establishing and preserving internal coherence of the conscious self-model, even if this process involves a retrospective confabulation.

One speculative empirical prediction following from these abstract considerations is that one function of mind wandering may be “autobiographical self-model maintenance,” the stabilizing of a functional platform that, first, causally enables the episodic activation of an EAM and, second, creates an adaptive form of self-deception, namely, an illusion of personal identity across time. That is, mind wandering is not only involved in automatic autobiographical planning (Baird et al., 2011; Mooneyham and Schooler, 2013; Stawarczyk et al., 2013) and the restructuring of a person's goal hierarchy (Klinger, 2013) *per se*, but also in the constant creation and functional maintenance of the representation of transtemporal continuity, a fictional “self” that then lays the foundation for important achievements like reward prediction, delay discounting, etc. (Redshaw and Suddendorf, 2013; Smallwood et al., 2013). Here, my conceptual point is that only if an organism simulates itself as being *one and the same* across time will it be able to represent reward events or the achievement of goals as happening to the same entity, as a fulfillment of its *ow*n goals^9^. Call this the “Principle of Virtual Identity Formation:” Many higher forms of intelligence and adaptive behavior, including risk management, moral cognition, and cooperative social behavior, functionally presuppose a self-model that portrays the organism as a single entity that endures over time. Because we are really only cognitive *systems* with a certain degree of physical and psychological continuity, but without any precise identity criteria, identity formation can only be achieved on a virtual level, for example through the creation of an automatic narrative. This could be the more fundamental and overarching computational goal of mind wandering, and one it may share with dreaming (Pace-Schott, 2013). Therefore, apart from the many specific adaptive functions it may have (Mason et al., 2013; Mooneyham and Schooler, 2013; Smallwood and Andrews-Hanna, 2013), mind wandering would then be a sort of baseline activity serving to maintain a minimal level of arousal and functional continuity, the default mode of autobiographical self-modeling, a permanent mechanism of re-encoding and synaptic stabilization, constructing a *domain-general* functional platform enabling long-term motivation and future planning. It is not M-autonomy itself that has this function. On the contrary, some types of mind wandering may actually be causally enabling factors in constituting the kind of conscious self-model an organism needs in order to episodically realize the functional property of M-autonomy plus the illusion of transtemporal identity (see section The Re-appearance of Meta-Awareness).

### Interim conclusion 1: mental autonomy as an exception

In conclusion, mind wandering can be understood as a loss of M-autonomy, because it involves an unnoticed loss of mental self-control and epistemic agency, either on the level of attention or of cognition. As an unintentional form of mental behavior it is not rationally guided, and while it is unfolding it cannot be terminated at will. Mind wandering is a failure of causal self-determination on the level of mental content, and although it clearly has aspects that can be described as functionally adaptive, its overall performance costs and its negative effects on general, subjective well-being are obvious and have been well documented (for example, in terms of reading comprehension, memory, sustained attention, or working memory, cf. Mrazek et al., 2013; Mooneyham and Schooler, 2013; Table 1). It is an important contribution of research on mind wandering to have demonstrated the ubiquity of the phenomenon and its effects.

We know that conscious mind wandering is a process that can get completely out of control (Schupak and Rosenthal, 2009; Bigelsen and Schupak, 2011), but that can also come completely to rest, either in practitioners of mindfulness meditation (Slagter et al., 2011; Mrazek et al., 2012) or following lesions to the medial frontal cortex (Damasio and van Hoesen, 1983). Under normal conditions, we spend 30–50% or our conscious waking lives mind wandering (Kane et al., 2007; Killingsworth and Gilbert, 2010; Schooler et al., 2011). During these times we do not possess M-autonomy. If we assume a 16-h day period, 40% of waking mind wandering would amount to an average of 384 min, a period during which we are not autonomous mental subjects. NREM-sleep mentation and non-lucid dreaming clearly are also periods during which the functional property of M-autonomy is absent, although complex cognitive processes are taking place across all sleep stages (Nielsen, 2000; Fosse et al., 2001; Fox et al., 2013; Wamsley, 2013; Windt, 2014) and can be sampled, for example using a serial awakening paradigm (Noreika et al., 2009; Siclari et al., 2013). Although great progress has recently been made in isolating the neural correlates of dream lucidity (Voss et al., 2009; Dresler et al., 2012) and developing a more fine-grained conceptual taxonomy for different kinds of lucidity (Noreika et al., 2010; Voss et al., 2013; Windt, 2014) it remains clear that M-autonomy during the dream state is a very rare phenomenon.

There are certainly phenomenological differences in reports following awakenings from REM sleep, which often result in elaborate narratives including complex multimodal imagery and intense emotions, and often less vivid and more thought-like reports from NREM sleep (Hobson et al., 2000; 799–803; Wamsley and Antrobus, 2009; for a comprehensive discussion see Windt, 2014, chapter 2); and there is clearly complex cognitive phenomenology during sleep as well (which also includes volitional components, Dresler et al., 2013); but as the frequency of lucidity (Metzinger, 2003a; Noreika et al., 2010; Voss et al., 2013) is generally so low as to be negligible for the present discussion, conscious thought during sleep can be treated as lacking M-autonomy almost entirely. Adults spend approximately 1.5–2 h per night in REM sleep (Hobson, 2002, pp. 77–79f). NREM sleep yields similar reports during stage 1, other stages of NREM sleep are characterized by more purely cognitive/symbolic mentation. Clearly, conscious thought during NREM-sleep also lacks M-autonomy, because it is mostly confused, non-progressive, and perseverative. Whereas 81.9% of awakenings from REM sleep yield mentation reports, the incidence of reports following NREM awakenings lies at only 43% (Nielsen, 2000, p. 855). If we assume an average REM-time of 105-min, there will be an average of 86 min characterized by phenomenally represented, but subpersonal cognitive processing; 375 min of NREM sleep will yield roughly 161 min of conscious mentation without M-autonomy. Assuming a waking period of 960 min, a very rough, first-order approximation is that human beings enjoy one sort of phenomenology or another for about 20 h a day (1207 min; or about 84% of their daytime), but healthy adults are only M-autonomous for 9.6 h (576 min; or 40% of an average day). These are very conservative estimates. For example, they also exclude life-time periods of illness, intoxication, or anaesthesia. In addition, there is evidence for extended periods in which human beings lose M-autonomy altogether. These episodes may often not be remembered and also frequently escape detection by external observers, as in “mind blanking” (Ward and Wegner, 2013). The same may also be true of periods of insomnia, in which people are plagued by intrusive thoughts, feelings of regret, shame, and guilt while suffering from dysfunctional forms of cognitive control, such as thought suppression, worry, rumination, and counterfactual imagery (Schmidt and van der Linden, 2009; Gay et al., 2011; Schmidt et al., 2011). We do not know when and how children actually acquire the necessary changes in their conscious self-model (Redshaw and Suddendorf, 2013), but we may certainly add the empirically plausible assumption that children only gradually acquire M-autonomy and that most of us likely lose it toward the ends of our lives.

The first conclusion to be drawn from this is that, according to our preliminary working concept of M-autonomy, human beings, although phenomenally conscious, are not autonomous mental subjects for roughly two thirds of their lifetime. A second, related conclusion is that conscious thought primarily and predominantly is an automatic subpersonal process, like heartbeat or immune autoregulation—and that on the conceptual level, we should do justice to this fact. It is empirically plausible to assume that a considerable part of our own cognitive phenomenology simply results from a frequent failure of executive control (McVay and Kane, 2009, 2010). I would claim that this actually is one of the most important functional and phenomenological characteristics of human self-consciousness, as a matter of fact, one of its most general, principal features: The almost constant presence of subpersonal and automatically generated mental activity as generated by certain parts of the default-mode network; (Raichle et al., 2001; Buckner et al., 2008; Mantini and Vanduffel, 2012), in combination with a frequent inability of the executive-control system to shield primary-task performance off against interference from these subpersonal thought processes (Smallwood et al., 2012). If I am right, autonomous cognitive self-control is an exception, not the rule.

Consequently, we will also have to depart from the “Myth of Cognitive Agency,” which says that the paradigmatic case of conscious cognition is one of autonomous, self-controlled rational thought. Hard-thinking philosophers, in particular, have perpetuated this myth like a phenomenologically self-fulfilling prophecy. Of course, it is always possible that their introspective experience really does differ, however, slightly, from that of the general population. But it is also possible that in thinking about these issues, they have mistaken exceptional cases of directed, effortful thinking of the type required for philosophical theorizing for paradigm cases of conscious mental activity. As M-autonomy has been intimately related to the professional self-understanding of philosophers for centuries there was a strong incentive, and clearly, we do, as an academic group, pride ourselves in being particularly good at it. But even if some of us may occasionally succeed in realizing these rationalist ideals, we probably only do so intermittently, and it is a merit of empirical research on mind wandering to finally make it clear that this type of controlled, effortful thinking may actually be a very bad model of conscious thought in general. The interesting question now becomes what it is that, sometimes, *makes* a subpersonal process into something that permits the ascription of personal-level psychological predicates.

## The distinction between subpersonal and personal processes

### The conceptual distinction

There are many levels of description on which we can gain knowledge about a human being. We can discover psychological truths about a given individual, but also biological (Millikan, 1984), or neurocomputational truths (Churchland, 1989; Clark, 1989; Hohwy, 2013), as well as physical truths about its bodily constitution. Which level we choose depends on our epistemic interests (what exactly it is that we want to know) and on a set of abstract methodological principles (for example, parsimony, coherence with existing theories, predictive power, heuristic fecundity). Often, there are types of explanation corresponding to such levels of description, and sometimes they stand in conflict.

Philosophers of mind have long distinguished personal and subpersonal-level explanations (Dennett, 1969, p. 93pp.; Davidson, 1980; Dennett, 1987). There have been extended technical discussions about whether one can speak of personal/subpersonal events, processes, states or facts in the same manner (Drayson, 2012), but also about the constitutive relevance of facts about subpersonal states for personal-level phenomena (McDowell, 1994; Hornsby, 2000; Colombo, 2012). Personal-level explanations are *horizontal*: they proceed from the past to the future in explaining single events by representing a diachronic causal relation as a horizontal line (Kim, 2005, p. 36). Subpersonal explanations are typically *vertical*: they concern the relationship between micro- and macro-levels, for example by explaining the abilities or dispositions an organism has in terms of its parts and their causal relations, or by giving a functional analysis that becomes more and more fine-grained. A well-known mistake is the ascription of psychological predicates to parts of a person's brain (e.g., “The prefrontal cortex plans actions”; “The premotor cortex decides on the initiation and organization of own movement sequences”). The conceptual mistake of ascribing a property that can only be ascribed to the whole entity to a part of it (called the “mereological fallacy”; see Bennett and Hacker, 2003, p. 72) often, but not necessarily accompanies the explanatory error of ascribing mental properties to subpersonal explananda (the “homunculus fallacy”; for a lucid discussion, see Drayson, 2012, section Is Conscious thought a Personal-Level Process?).

Interestingly, the concept of *wandering* has personal and subpersonal uses at the same time, even groups of persons or physical objects engage in it. Philosophers engage in peripatetic wandering, some of them become wandering monks or even wander from the path of righteousness by becoming wandering preachers, nomadic tribes wander, etc.—but so do bullets, kidneys, testicles, the poles or the Earth's magnetic field. In current publications about mind-wandering, the use of the term “mind” subtly oscillates between personal and subpersonal uses as well. “Mind” is a folk-psychological term, but it is often used as if referring to a unitary entity that “does” the wandering (e.g., “… lapses in “control” allow the mind to escape from what it is doing,” cf. Smallwood et al., 2012, p. 67, Box 3). Therefore, an important future step will be to clearly acknowledge the fact that mind wandering is a target for genuinely vertical, subpersonal psychological explanation, and to dissolve the vague, folk-psychological umbrella term “mind” into a well-ordered set of successor concepts.

### Is conscious thought a personal-level process?

In some of its aspects, gaining meta-awareness of ongoing mind-wandering is like gaining meta-awareness of your breath or your heartbeat. Heartbeat, breathing, and seemingly task-unrelated, phenomenologically spontaneous thoughts are not personal-level psychological processes that are mysteriously correlated with or caused by some physical chain of events. The most parsimonious metaphysical interpretation of the relevant scientific data is that they are *identical* with functionally complex, but subglobal physiological processes in the biological body. In the case of momentary lapses of attention and mind-wandering, this physiological process is a specific, widely distributed pattern of neural activity, and it is now empirically plausible to assume that large parts of this pattern overlap with activity in the default mode network [DMN; (Weissman et al., 2006; Buckner and Carroll, 2007; Mason et al., 2007; Buckner et al., 2008; Christoff et al., 2009; Spreng et al., 2009; Andrews-Hanna et al., 2010; Stawarczyk et al., 2011; Christoff, 2012; Mantini and Vanduffel, 2012; Gruberger et al., 2011)]. Any rational research heuristics for heartbeat, breathing, or the critical subset of DMN activity will therefore treat them as subpersonal, bodily processes. All three of them have a long evolutionary history (Lu et al., 2012; Mantini and Vanduffel, 2012; Corballis, 2013), and all three of them are clearly dynamic, self-organizing chains of events that continuously and automatically unfold over time, and not agentive processes implying explicit goal-selection, rationality constraints, etc. If one adds the straightforward metaphysical assumption of a domain-specific identity (McCauley and Bechtel, 2001; Bickle, 2013) holding between the phenomenal states constituting episodes of mind wandering and what we are currently beginning to discover as their local, minimally sufficient neural correlates (Chalmers, 2000), then it becomes clear that mind wandering simply is the phenomenal awareness of a local bodily process^10^.

### A sketch of a model to solve the interface problem

But why do we subjectively experience some of our cognitive processes *as* personal-level properties from the first-person perspective? There is a long story to be told here (Metzinger, 2003a, 2006, 2007, 2008), but the short answer is this: Because they have been embedded into an EAM, which is currently active in our brain; and because we live in a normative sociocultural context in which we folk-psychologically describe and reciprocally acknowledge each other as rational individuals, which in turn influences introspective experience itself. I will not go into any of the theoretical complications here, but will briefly sketch an oversimplified model of the transition from subpersonal to personal-level states. The philosophical problem in the background is the question of how precisely common-sense psychological explanation interfaces with the explanations given by scientific psychology and cognitive neuroscience (Bermúdez, 2005, section Interim Conclusion 2: Conscious thought as a Subpersonal Process). My point is that the conscious brain—which had to predict the organism's complex internal dynamics (Seth et al., 2012) as well as its external behavior on a global, whole-organism scale (Metzinger, 2014)—solved this problem long before psychologists and philosophers even conceived of it in the first place. What it needed was a flexible instrument allowing the system to *appropriate* some of its subpersonal processes on the representational level, as well as on the level of causal self-control. We have to understand the connection between subpersonal and personal-level *phenomenology* in order to understand the intuitions that create the methodological and philosophical issues constituting the interface problem.

The decisive component is a specific kind of conscious self-representation, a PSM (see section Making Progress on the Phenomenology of Mind-Wandering below) that portrays the system as an epistemic agent, as an entity that is actively searching for and optimizing its knowledge, for example by controlling its own high-level, quasi-symbolic processing as a CA or by actively sustaining and controlling the focus of attention (AA). This is what I call an EAM^11^. It can be shown that human beings can enjoy a minimal form of self-consciousness without possessing an EAM (Blanke and Metzinger, 2009; Limanowski and Blankenburg, 2013). The transition from simple, bodily self-identification to this much stronger form takes place when a system phenomenally represents itself as an entity capable of epistemic agency, or even as one currently exerting epistemic agency. If such a specific kind of self-model is in place, ongoing processes can be *embedded* into it, thereby creating the phenomenology of ownership (*my* thought, *my own* autobiographical memory, *my own* future planning). If these processes are represented as control processes, as successful acts of exerting causal influence, they can now be consciously experienced a processes of *self*-control or successful mental *self*-determination. Yet, an epistemic-agent model of this kind is not a little man in the head, but itself an entirely subpersonal process. Human beings only *become* persons by having the potential to phenomenologically identify with the content of such a model (see section Mind Wandering as a Switch in the Unit of Identification), a step which on the sociocultural level causally enables practices like linguistically ascribing person-status to themselves and mutually acknowledging each other as subjects of experience, epistemic agents, and rational individuals.

### Interim conclusion 2: conscious thought as a subpersonal process

The first interim conclusion was that human beings, although phenomenally self- conscious, do not enjoy M-autonomy for roughly two thirds of their conscious lifetime. This yields a simple, quantitative argument for slightly reorganizing the conceptual landscape by letting go of the “Myth of Cognitive Agency” as the implicit, paradigmatic model of what conscious cognitive processing really is. In addition, it is now empirically plausible to hypothesize that the neural correlate of non-autonomous conscious thought overlaps to a considerable degree with ongoing activity in the DMN, therefore the postulation of a local, domain-specific identity is a tenable, coherent metaphysical interpretation of this fact. Whatever will figure as the explanans in a future scientific theory of mind wandering will therefore not be global properties of “the mind” or the person as a whole, but specific microfunctional properties realized by the local neural dynamics underlying each episode of consciously experienced subpersonal cognitive processing. It remains true that a considerable part of conscious thought (very roughly, one third) is experienced as CA from the first-person perspective and consequently ascribed as a personal-level property. Therefore, a second major target for future research is to investigate the *transitions* connecting single episodes of mind wandering with episodes of M-autonomy or immediately following further episodes, and those events that we currently describe as the regaining of meta-awareness (Schooler et al., 2011).

## Making progress on the phenomenology of mind-wandering

A useful conceptual instrument to develop more fine-grained descriptions of the phenomenology of mind-wandering is the notion of a PSM; (Metzinger, 2003a, 2006, 2007). A PSM is a conscious representation of the system as a whole, including not only global body representation (Blanke and Metzinger, 2009; Metzinger, 2014), but also psychological, social and other potential personal-level properties. One central idea of the self-model theory (Metzinger, 2003a) is that, under standard conditions, a large part of the human PSM is “transparent,” because we are not able to experience it *as* a model and therefore fully identify with it representational content.

In order to describe the phenomenology of mind wandering more precisely, it is a good strategy to have a closer look at the onset of an episode as well as at the way in which periods of mind-wandering end, when we suddenly “come to ourselves” again. What is needed are individuation criteria telling us what exactly *one* episode is, and how its temporal boundaries can be determined. In the following three sections I will sketch three ways in which one might gain fresh perspectives on mind wandering by focusing on the onset and the termination of mind wandering episodes plus what I take to be the perhaps most interesting, but hitherto neglected phenomenological characteristic of the two transition phases marking the onset and the end of each episode: The sudden *shift in the unit of identification* (UI).

### The self-representational blink

If we conceive of the process that “switches” between perceptually coupled states and states that are decoupled from the current situation as a dedicated functional module, then this module must employ a set of criteria, which are as yet unknown (Klinger, 2013, p. 14). However, the human brain can also be viewed as a physical system that fluidly, rapidly, and continuously undergoes metastable transitions between different dynamical states, for example those characterizing the mind wandering state and those characterizing M-autonomy and meta-awareness (Bressler and Kelso, 2001). An important aspect of the phenomenology of mind wandering is that no such criteria or dynamical constraints are explicitly represented on the level of the PSM (Metzinger, 2003a, 2007): We do not know *why* an episode of mind wandering has just begun, and the very first content element typically comes as a surprise. We may sometimes possess what Schooler et al. (2011) call “meta-awareness” for the second “carriage” in a train of thought, as it were, but the appearance of the first content element, subjectively, is an unpredicted event, thus, contributing to the subjective sense of lost control and the sudden appearance of unintentional mental behavior. We may think of this as a systematic blindness of autobiographical self-representation, on a very short time-scale: The dynamical shift itself, the actual event of transition into the mind wandering state is not something we can remember, it is not part of our conscious mental life.

Let me introduce a new technical term to refer to this phenomenon, the “self-representational blink” (SRB). Phenomenologically, the SRB is characterized by a brief loss of self-awareness, followed by an involuntary shift in the unit of identification (see next section). Functionally, we can describe it as a failure of attentional and/or cognitive self-control, perhaps as a depletion of resources. Alluding to the now well-studied phenomenon of the attentional blink (Raymond et al., 1992; Shapiro et al., 1997), the notion of a “self-representational blink” refers to the fact that we are typically not able to consciously experience the actual moment of transition from mindful, present-oriented self-awareness to the identification with the “protagonist” of a daydream, the content of the self-model in autobiographical planning, depressive rumination, etc. Here, the speculative hypothesis developed from conceptual considerations is that if the *mechanism of temporal self-location* has to be briefly suspended and two different PSMs follow in close temporal proximity, the first one will be easy to identify, remember, and report, but performance on the contents of the second target will be impaired. Therefore, the empirical prediction is that subjects should be blind to self-related stimuli during the SRB. “Self-related stimuli,” for example, are all stimuli that have to do with current body-perception, like interoceptive, visceral sensations, nociception, vestibular input, proprioception, thermal and gravitational self-perception, etc. More generally, self-related information is all information that can in principle be represented in a cognitive system's PSM. The SRB would be the brief temporal window separating the PSM of the last conscious moment in which the system was still perceptually coupled to the current environment from the rapidly following PSM that constitutes the subjective center of a mind wandering episode, or the conscious self of the daydream. Within this window, there would be self-blindness on the level of phenomenal representation.

We do not know what the neural mechanisms are that allow human beings to mentally locate themselves in a temporal order, to define the point in time at which the self-as-represented is currently situated, and we also do not know the mechanisms underlying *virtual* temporal self-location as in “mental time travel” (Buckner and Carroll, 2007). However, ongoing research on isolating the minimal conditions for phenomenal self-consciousness increasingly converges on the notion that transparent spatiotemporal self-location is necessary and sufficient in order to create a robust sense of self, with ongoing attentional and CA not being necessary conditions (Blanke and Metzinger, 2009; Windt, 2010, 2014; Metzinger, 2013a). The onset of a mind-wandering episode clearly is a shift from one PSM to the other, and while a weak sense of spatial situatedness and embodiment are preserved, self-identification at a specific moment in time is often broken and discontinuous. Mind wandering is “involuntary mental time travel” (Song et al., 2012). This discontinuity in temporal self-modeling interestingly resembles the frequent transitions found in the dream state (Windt and Metzinger, 2007), and it might also explain the phenomenology of the SRB. It seems to be a conceptually necessity that, in order to shift from real-time/real-world self-modeling (as anchored in the here and now, and ultimately defined by the spatiotemporal position of the physical body) to the virtual self-representation of a daydream or of autobiographical planning (Stawarczyk et al., 2013), the system has to achieve a dynamical transition departing from the subpersonal process of temporal self-location that co-constitutes the previous minimal self-model. In standard cases, there will be a discontinuity, because the current temporal frame of reference is substituted by a new one^12^. If this speculative hypothesis, which is derived from conceptual considerations plus the description of a specific phenomenological aspect alone, points in the right direction, it should be possible to investigate the functional transition underlying the SRB experimentally (McVay and Kane, 2013).

### Mind wandering as a switch in the unit of identification

Let us look at a second phenomenological feature of mind wandering that could, if correctly described, yield a new theoretical perspective. Perhaps the most interesting phenomenological feature of mind wandering is a sudden shift in the UI. The UI is the phenomenal property with which we currently *identify*, exactly the form of currently active conscious content that generates the subjective experience of “I *am* this!” Please note how many mind wandering episodes are phenomenologically disembodied states, because perceptual decoupling (Schooler et al., 2011) often also means decoupling from current body perception (Metzinger, 2009, 2014; but see Miles et al., 2010a,b). A typical phenomenal property serving as the “target” of the hypothetical and as-yet unknown mechanism of self-identification (Blanke and Metzinger, 2009) would be the integrated contents of our current body image, another standard example is the subjective quality of “agency” in the control of bodily actions. Often, both target properties coincide and simultaneously function as the locus of identification. This is why, in standard situations, we experience ourselves as embodied agents. *Prima facie* one might think that the phenomenal self simply is wherever there is an experience of causal control, for example in bodily or mental agency. The phenomenally experienced “sense of effort” in bodily and mental action would then be the essence of self-consciousness. However, data about asomatic OBEs and bodiless dreams show a more differentiated picture: For example, human beings are able to passively identify with a non-extended point in space only while retaining a robust sense of self (De Ridder et al., 2007; Windt, 2010; Metzinger, 2013a). The interesting contribution research on mind wandering now makes is that even during ordinary wake states, there are frequent and uncontrolled shifts in the UI, and that these shifts are not deliberately initiated personal-level events, but causally determined by unconscious, and as yet not understood, processes on the subpersonal level of description. After each of these shifts, the sense of mental agency is lost, and often the conscious sense of embodiment is considerably weakened as well (Metzinger, 2014).

What remains when we lose M-autonomy and fall into a mind wandering episode? It is a low-resolution *situation model*, including an integrated representation of the body as located in a spatial frame of reference, i.e., a model of the environment plus an embedded bodily self. This default situation model keeps the immediate, overall perceptual context available for selective attention and simply does what it always does: it automatically and continually minimizes prediction error by monitoring body and immediate environment for potential unexpected events (Friston, 2010; Mantini and Vanduffel, 2012, p. 84; Hohwy, 2013; Limanowski and Blankenburg, 2013; Seth, 2013). However, during the mind wandering state, the system does not engage in active sampling or precision optimization relative to its current physical environment. This activity has now shifted from the real to the virtual world. What is lacking is a stable subject-object-structure in the sense of a knowing or acting self as *directed* toward specific target objects in the proximal environment or the current interoceptive body landscape, an EAM (see section Mental Autonomy). The interesting point is that the process of temporal self-location is now partly dissociated from the process of spatial self-location: The UI shifts to the protagonist of our current mind-wandering episode, say, the model of a future self as employed in periods of autobiographical planning. When not mind wandering, spatial self-location, temporal self-location, and self-identification (Blanke and Metzinger, 2009) coincide, during mind wandering episodes they become functionally dissociated in an interesting way. Just like the functional mechanism of temporal self-location, the subpersonal mechanisms leading to the phenomenology of “I *am* this!” are clearly open to experimental investigation (Lenggenhager et al., 2007; Blanke and Metzinger, 2009; Metzinger, 2013a; see Blanke, 2012 for review).

Given the new conceptual tool of a UI, two important further phenomenological constraints for any future theory can be described more clearly. First, there can be rapid, cyclically recurring switches between two or more UIs. This can for example be the case in the neurological condition of heautoscopy, where the subject experiences seeing a second own-body in extracorporeal space (Lukianowicz, 1958; Menninger-Lerchenthal, 1961; Brugger et al., 1997; Brugger, 2002; Blanke and Mohr, 2005; Blanke, 2012, p. 562), or during situations where we quickly alternate between mind wandering and briefly returning to the real-world task at hand, or to a cognitive task that demands CA and AA. In heautoscopy, self-location may frequently alternate between different embodied and visually hallucinated extrapersonal positions and may even be experienced at two positions simultaneously. In mind wandering, we also frequently oscillate between two UIs, for example the virtual conscious self of our daydream or depressive rumination, and the perceptually coupled PSM that is needed for selective, flexible, and context-sensitive motor control, to briefly return to the current task in the real world. The second constraint is that there clearly is a variable *strength* in the degree of identification. In mind wandering, there is a phenomenological “depth of immersion” that is in need of explanation and should be amenable to further experimental investigation (Schad et al., 2012), and perhaps it may be related to the cognitive system's constant task to find and intelligent trade-off between external and internal resource distribution (Thomson et al., 2013).

### The re-appearance of meta-awareness

How exactly does an episode of mind wandering end? Schooler and colleagues, referring to work by the late Daniel Wegner, point out that regaining meta-awareness may be accompanied by an illusion of control (Schooler et al., 2011, Box 1; Wegner, 2002). Whenever we have this case, it seems that a specific new self-model has appeared: An autobiographical self-representation depicting the last mental event as something that was self-controlled, an instance of deliberate causal self-determination on the mental level. This form of control is often described as an *autoepistemic* form of self-control, as an instance of actively acquired self-knowledge or a sudden insight. Thus, a typical autophenomenological report may claim “I have just regained meta-awareness, because I just introspectively realized that I was lost in mind wandering!” Do we have reason to believe such claims? Is the reappearance of meta-awareness a subpersonal event or is it something in which global control and the conscious EAM actually played a decisive causal role?

Because mindfulness and mind wandering are opposing constructs (Mrazek et al., 2012), the process of losing and regaining meta-awareness can be most closely studied in different stages of classical mindfulness meditation (Hölzel et al., 2011; Slagter et al., 2011), In the early stages of object-oriented meditation, there will typically be cyclically recurring losses of M-autonomy, plus an equally recurring mental action, namely the decision to gently, but firmly bring the focus of attention back to the formal object of meditation, for example to interoceptive sensations associated with the respiratory process. Here, the phenomenology will often be one of mental agency, goal directedness, and a mild sense of effort. In advanced stages of open monitoring meditation, however, the aperture of attention has gradually widened, typically resulting in an effortless and choiceless awareness of the present moment as a whole. Such forms of stable meta-awareness may now be described as shifts to as state *without* a UI^13^. Whereas in beginning stages of object-oriented mindfulness practice, the meditator identifies with an internal model of a mental agent directed at a certain goal-state (“the meditative self”), meta-awareness of the second kind is typically described as having an effortless and non-agentive quality. Interestingly, the neural correlates pertaining to this difference between “trying to meditate” and meditation are now beginning to emerge (Garrison et al., 2013).

Schooler and colleagues define meta-awareness as “one's explicit knowledge of the current contents of thought” (2011, p. 321). First, this raises the question what exactly an “explicit” representation is, as opposed to an “implicit” representation. Our intuitions about what makes a representation explicit are inconsistent (Kirsh, 2006, p. 345; Palmer, 1978). “Explicit” could mean topological equivalence between representational content and representandum (e.g., in a perceptual object representation, parts of the perceived object could be directly mapped onto parts of its corresponding neural representation), it is often vaguely equivocated with “conscious,” or it could imply locality, syntactic compositionality, semantic transparency (Clark, 1989), or perhaps just mean “symbolic,” in the sense of “conceptual,” or “propositional.” If I simply attend to the process of mind-wandering, without forming a mental concept or engaging in any form of mental judgment, memory, or categorization (Hölzel et al., 2011)—would this be an explicit or an implicit form of mental meta-representation? Would it be meta-awareness? In this situation, I would possess M-autonomy and would therefore be able to terminate the first-order process of mind-wandering at will; I would satisfy the AA-constraint (I would be an attentional agent), but I would only satisfy the CA-constraint in a weaker sense (because I would not be engaging in high-level, symbolic thought, although I would possess the corresponding ability and know about this fact).

A second aspect of Schooler et al.'s proposed definition that calls for future differentiation in the important notion of an individual “noticing” the current contents of their mind (Chin and Schooler, 2009; Schooler et al., 2011, Box 1) is the conflation of knowledge and phenomenal experience. Postulating “explicit *knowledge* of the current contents of thought” (Schooler et al., 2011, p. 321; emphasis TM) excludes the possibility of higher-order misrepresentation, of being wrong about the current contents of one's own mind: In regaining meta-awareness, we might sometimes misrepresent the contents of our own mind (without being able to notice the meta-cognitive deficit itself), or we might even hallucinate first-order mental content that was never there in the first place. What we subjectively *experience* as a form of knowledge does not have to be knowledge. Conceptually “meta-awareness” (a phenomenological notion) is not a “subtype” (Chin and Schooler, 2009, p. 33) of meta-cognition (an epistemic notion). If meta-awareness is supposed to be explicit in the sense of quasi-symbolic mental representation, then an independent epistemic justification would be needed to call it knowledge. In short, defining the end of a mind-wandering episode as a form of introspective knowledge buys one into a host of epistemological problems that have plagued philosophers for a long time. It may therefore be better to confine empirical research programs to the phenomenology of mind wandering.

The concept of “mind wandering” was originally introduced into debate as a *phenomenological* notion (Smallwood and Schooler, 2006), i.e., it has been treated as a process that is available for introspective attention and verbal report, a specific kind of subjective experience (Seli et al., 2013). Therefore, concepts like “stimulus-unrelated thought” (SIT; e.g., Smallwood and Schooler, 2006) or “stimulus-independent and task-unrelated thought” (SITUT; Stawarczyk et al., 2012) are only *phenomenological* terms as well: We may subjectively experience the first event in a conscious train of thought as unpredictable, uncaused, and not goal-directed. And indeed, research on mind wandering has already yielded a whole range of interesting results concerning the phenomenology of thought, which, for example, are highly relevant for the predominantly philosophical debate on “cognitive phenomenology” and could be interestingly related to the current philosophical discussion on this topic (Bayne and Montague, 2011). However, this contingent fact does not exclude the logical possibility of unconscious mind wandering: What we can consciously access as daydreaming, inner thoughts, fantasies, and feelings may rather be just the tip of the iceberg, a small partition of a much larger state space in which the continuous cognitive dynamics unfolds. Conscious mind wandering would then be characterized by a higher degree of coherence, but still emerge out of a larger unconscious background of activity (Horovitz et al., 2009; Vanhaudenhuyse et al., 2010; Samann et al., 2011).

However, as mind-wandering plausibly is identical with a process in the brain, and as one fundamental principle of science is the assumption of the “Causal Closure of the Physical” (stating that every event that has a cause has a physical event as its sufficient cause, cf. Kim, 1993, 2005; Stoljar, 2009), the “spontaneity” of the onset of mind wandering is only a phenomenological property as well (and one that can perhaps be scientifically explained via the SRB, see section The self-representational blink). There will not only be unconscious neural precursors of mind wandering itself, but also specific, introspectively inaccessible goal representations that drive the high-level phenomenology of mind wandering (Klinger, 2013), for example, of postponed goal-states which have been environmentally cued by goal-related stimuli under high cognitive load (Cohen, 2013; McVay and Kane, 2013), as well as unconscious causal antecedents for 2nd-order acts of intentionally *inhibiting* the flow of activity (Filevich et al., 2013). There is no free *won't*. From a functional perspective, M-autonomy dramatically expands our inner and outer space of possible behaviors, it is an entirely new level of naturally evolved intelligence. But this fact does not imply a libertarian concept of free will assuming uncaused, but causally effective mental events. The PSM *can* be involved in the process of self-control by enabling veto-style inhibition and a phenomenology of top-down control, but it does not have to be. Successful inhibition can be an entirely subpersonal process. For example, it has been demonstrated that inhibitory control processes can be modulated in a completely unconscious and unintentional manner (Hepler and Albarracin, 2013) and that unconscious no-go stimuli are sufficient to activate prefrontal control networks in the inferior frontal cortex and the pre-supplementary motor area (van Gaal et al., 2010). This also means that “task-unrelatedness” is a phenomenological notion only: It subjectively *appears* to us as if such episodes were not goal-directed, but given an unconscious functional context, they may well be adaptive and contribute to task solution (Klinger, 2013; Mooneyham and Schooler, 2013). In addition, a well-known fact from perceptual psychology is that the discriminatory resolution power of attention outstrips category formation (for a philosophical discussion, see Raffman, 1995). Consequently, there may be much more to the phenomenology of mind wandering than is verbally reportable—simply because introspective attention is much more subtle and nuanced than the type of conceptually mediated cognitive access leading to verbal report.

In an important theoretical paper Jonathan Smallwood (2013; for critical discussion see Franklin et al., 2013) has introduced the distinction between those functional processes that govern the occurrence of a conscious thought and those that control the way in which it later begins to unfold over time. This view can be interestingly integrated with the self-model theory of subjectivity (Metzinger, 2003a,b, 2006, 2007, 2008), which assumes the existence of an unconscious as well as a conscious self-model. The conscious self-model (or PSM) is that partition of a more comprehensive global self-representation that is functionally available for introspective attention, top-down cognitive control, and the generation of flexible, context-sensitive behavior. Given this framework, there can be unconscious goal-representations (as dynamic parts of the unconscious self-model) that trigger extended unconscious cognitive processes, which at some point become integrated into the PSM. As only the content of the conscious self-model is available for attention and verbal report, the resulting subjective phenomenology would often be one of spontaneity and lack of goal-directedness. Sincere first-person reports, necessarily, would then often describe such processes as “task-unrelated.” From a third-person perspective, however, it is obvious that a complex human organism constantly has to solve *many* problems at the same time, in different time windows, with multiple goal-states to be achieved, some of them conflicting and/or lying in the distant future, and that most of these subpersonal, long-term problem-solving processes will take place in the unconscious self-model. Whenever such parallel processes spill over from the unconscious into the conscious self-model, new forms of control like intentional inhibition or attentional modulation become available. However, the person herself might not consciously recognize the relevance of the corresponding conscious thought content at all, simply because its relation to her own long-term goals plus a preceding, and possibly extended, processing history are only represented in the unconscious self-model.

Franklin et al. (2013, p. 540) identify determining the “when” of mind wandering as “the major obstacle to further significant advances in mind wandering research” while pointing out potential confounds between the frequency of mind wandering and the duration of single episodes. Here, it is important to note that the individuation of mental events is itself a difficult conceptual issue. At the very least, one must distinguish between the *phenomenological individuation* of mental events via subject's reports (i.e., by first-person introspection of PSM-content) and the *functional individuation* of events (i.e., by their independently observable causal role). Smallwood's process-occurrence framework may therefore help to isolate different functional components of thought, and one can perhaps describe the self-representational blink and the reportable onset of a mind-wandering episode as exactly those moments where some aspects of the massively parallel activity in the unconscious self-model spills over into the PSM. The “occurrence” would then be the (phenomenal) moment at which this activity can be introspectively detected for the first time, whereas the “process” is a (functional) chain of events that crosses the boundary between the unconscious and the conscious self-model, perhaps even multiple times and in both directions.

Let me close by pointing to a structural commonality with well-known problems in dream research, which may shed further light on the issue what exactly it means that a mind-wandering episode *ends*. First, there is the phenomenon of “false awakening,” that is of realistic dreams of waking up (Green, 1994; Windt and Metzinger, 2007; Windt, 2014); second, current research shows that there are different levels or stages of becoming lucid in a dream (Noreika et al., 2010; Metzinger, 2013a; Voss et al., 2013). If there is an additional awareness of meta-awareness as just having been regained (i.e., a third-order meta-representation or second-order EAM), then the point made in the previous paragraph also applies: As such, this is just phenomenal experience, and not necessarily knowledge—we might always be introspectively self-deceived. However, this purely conceptual point is interestingly related to the empirical problem of defining the end of specific episodes in research on mind-wandering: If “noticing” involves an explicit meta-cognitive self-representation in terms of categorizing one's own current thought contents, of applying a concept or making a mental judgment (“Oops, I have just been daydreaming again!”), this may often lead to yet another train of thought *about* having regained meta-awareness, M-autonomy, and so forth. If veto control, CA, and AA are lost in this process, then we may speak of a second mind wandering episode having just begun. There is now a new phenomenal UI (the phenomenal subject of meta-awareness or “meta-cognitive self”), but the functional properties characterizing M-autonomy are not realized by the brain.

How does one conceptually individuate mind wandering episodes, how does one turn them into countable entities? This is a methodological problem every good theory about mind wandering will have to solve: If mind wandering episodes are to be well-defined research targets and proper theoretical entities, one must able to exactly specify the identity criteria that make one such episode one and the same episode. If the existence of a SRB at the beginning (see section The Self-Representational Blink) is a necessary condition, and if we take the shift to a new UI (section Mind Wandering as a Switch in the Unit of Identification) as a second criterion for individuating episodes on mind wandering, then we arrive at an interesting result. There are at least two ways in which a mind wandering episode can end, one including the SRB plus a shift to a new UI, and one without a shift to a new UI.

## Conclusions

Research on mind wandering holds great promise for having substantial, long-lasting impact in many fields of philosophy at the same time, not only in philosophy of mind, cognitive science, and psychology, but also in epistemology, applied ethics, political philosophy, or philosophy of law. At the same time, empirical research can profit from conceptual clarifications, constructive methodological criticism, and the metatheoretical perspective offered by philosophers. I will therefore conclude by offering a non-exhaustive list of conclusions and desiderata for future research involving both disciplines.

- For about two thirds of their conscious lifetime, human beings do not possess “M-autonomy”: Rational mental self-control, the ability to terminate ongoing subpersonal mentation at will, or to actively establish individual goal-commitments and to impose rules onto one's own mental behavior, are comparatively rare phenomena. As the large majority of our mental activity is not driven by explicit, consciously available goal-representations and cannot, while it is unfolding, be inhibited, suspended or terminated, we are not mentally autonomous subjects for about two thirds of our conscious lifetime.
  ◦ The working concept of “M-autonomy” is a heuristic tool, which must be empirically grounded, semantically enriched, and continuously differentiated.
  ◦ The functional potential for M-autonomy can be operationally defined. Is it a good candidate for a novel criterion for personhood?
- On the level of conscious mental activity, epistemic agency is an exception, not the rule. For human beings, epistemic agency can be differentiated into (CA; the ability to control goal-directed/task-related, deliberate thought) and (AA; the ability to control the focus of attention). For most of their conscious lifetime, human beings are neither cognitive nor attentional agents, and they also lack an explicit phenomenal self-representation of themselves as currently *possessing* these abilities. Conceptually, most of our conscious activity must therefore be characterized as a form of unintentional mental behavior.
  ◦ Quantitative studies investigating the distribution of CA and AA over the whole sleep-dream-wake cycle more precisely are an important desideratum for future research.
  ◦ Philosophical action theory can now be interestingly expanded to mental actions, integrating a large number of novel bottom-up constraints delivered by psychological research on mind wandering.
- Empirically, it is now plausible to assume a large overlap between the minimally sufficient neural correlate of episodes of mind wandering and activity in the DMN. Therefore, mind wandering is a target for vertical, subpersonal psychological explanation. Those neurofunctional properties that constitute an episode of mind wandering and determine its phenomenal content, and which will prominently figure in a future scientific explanation, will not be global properties of “the mind,” or properties that have to be ascribed on the personal level of description.
  ◦ The vague, folk-psychological umbrella term of “mind wandering” should be dissolved into a well-ordered set of successor concepts.
  ◦ Mind wandering, daydreaming, depressive rumination, perserverative cognition, or intrusive thoughts in insomnia likely are all different phenomena (Ottaviani and Couyoumdjian, 2013). Therefore, the catalog of explananda should continuously be differentiated.
  ◦ The minimally sufficient neural correlates corresponding to each of the resulting theoretical constructs should be isolated.
  ◦ What exactly is the constitutive relevance (Colombo, 2012) of mind wandering for high-level cognition and the emergence of a first-person perspective?
- Roughly two thirds of conscious thought must be described as a subpersonal process that functionally results from a cyclically recurring loss of M-autonomy. A parsimonious metaphysical interpretation of available empirical data suggests a domain-specific identity of non-autonomous conscious thought with a large subset of DMN-activity. Conceptually, mind wandering is not a property of the person as a whole, but a set of functional properties realized by a specific part of the brain and its temporal dynamics.
  ◦ What are these properties, what is their evolutionary history?
  ◦ The logical relationship between personal-level and subpersonal explanations should be more closely investigated for the special case of conscious cognition.
- Internally, cognitive processing only *becomes* a personal-level process by being functionally integrated into and actively controlled with the help of a specific form of transparent conscious self-representation, the EAM. An important conceptual distinction is the one between conscious self-representation of ongoing cognitive or AA and the passive representation of the ability to act as an epistemic agent, involving the phenomenology of knowing about the potential for mental action without actually realizing it.
  ◦ In describing rapid fluctuations and gradual transitions between periods of meta-awareness and episodes of mind wandering, it may be helpful to distinguish a weak and a strong EAM.
  ◦ “Zoning out” or unaware mind wandering can be described as a complete collapse of the EAM, whereas “tuning out” would be the retaining of a conscious self-model involving the ability for epistemic agency, a weak EAM.
  ◦ What exactly is the causal contribution the EAM makes? Which of its aspects are purely phenomenal or of a confabulatory nature?
- The phenomenology of mind wandering must be described in a more fine- grained manner. Individuation criteria for individual episodes should be developed.
  ◦ What are the temporal boundaries of episodes of mind wandering?
  ◦ Does every episode begin with a self-representational blink? Can the hypothesized blindness to self-related stimuli be empirically verified?
  ◦ Can individual episodes successfully be categorized according to their UI? Can questionnaires be optimized in order to reliably pick out the UI, turning it into a new dimension for scientific taxonomies of subpersonal cognition?
  ◦ How can research on mind wandering help to describe the EAM in its purest form, including its collapse and re-emergence at the onset and end of each episode?
  ◦ Is what has been termed “meta-awareness” in the psychological literature necessarily a form of self-consciousness? Is there evidence for non-egoic forms of meta-awareness?
- Meditation is a systematic, formal practice of cultivating M-autonomy.
  ◦ How are attentional lapses, the SRB, and periods of M-autonomy distributed across different stages and types of meditation?
  ◦ In meditation, is there evidence for mind-wandering episodes that do not begin with a SRB?
  ◦ In what way does the ending of a mind-wandering episodes differ from the corresponding transition during ordinary everyday life? Which forms of emerging meditative meta-awareness can be conceptually described as a shift in the UI, which ones do not involve a new unit of identification?
  ◦ In mindfulness meditation, what is the quantitative relationship between mind wandering, meta-awareness (Schooler et al., 2011), and mind blanking (Ward and Wegner, 2013)?
  ◦ Are there systematic illusions of control involved in meditation practice, for example, when exiting an episode of mind wandering?
  ◦ How does the event of regaining meta-awareness in meditation differ from corresponding transitions in ordinary waking states and in the dream state?
- Interesting commonalities between the phenomena of mind wandering and nocturnal dreaming are increasingly beginning to emerge (Metzinger, 2013b).
  ◦ What exactly is the relationship between M-autonomy, the occurrence of different stages of dream lucidity and what researchers in mind wandering call “meta-awareness”?
  ◦ Can lucid lapses and mind-wandering lapses plausibly be interpreted as the disintegration of the EAM?
  ◦ Are there common *positive* functionalities connecting dreaming and mind wandering during wake states, such as the encoding of long-term memory, complex, preparatory motor planning, or creative incubation?
  ◦ False lucidity and the phenomenology of insight: In becoming lucid and in daytime mind wandering, is the experience of *oneself* having actively regained meta-awareness (and thereby M-autonomy) an illusion of control over a mental event that was really triggered by an unconscious process?
  ◦ All these topics are of direct interest to the philosophical project of “cognitive phenomenology.” In particular, the specific “phenomenology of insight” going along with becoming lucid in a dream or with successfully catching oneself mind-wandering in meditation may prove to be of central importance. In the future, a stronger connection between the philosophical debate on cognitive phenomenology and empirical research on mind wandering, meditation and lucid dreaming is desirable.

### Conflict of interest statement

The author declares that the research was conducted in the absence of any commercial or financial relationships that could be construed as a potential conflict of interest. This research was supported by the European FP7 collaborative project VERE (contract no. 257695).

## Footnotes

^1^“Cognitive phenomenology” is a new subfield of research in philosophy of mind that focusses on the phenomenal character of occurrent non-sensory mental states like thoughts or wishes, and on the distinct subjective quality that goes along with thinking (see Bayne and Montague, 2011 for a good overview). Some philosophers claim that there is a proprietary, distinctive, and individuative phenomenology of higher cognitive processing that cannot be derived from sensory phenomenology, others deny this claim. For present purposes, I leave this controversial issue to the side. My goal is more basic: namely to develop a taxonomy for describing conscious thinking.

^2^This means that AA is one specific example of having a consciously experienced first-person perspective (1PP). Its theoretical relevance consists in the fact that it is plausibly the simplest form of an EAM human beings can have. For philosophers, the concept of a 1PP is highly relevant, and for a wide range of reasons. For example, one classical philosophical issue is the question of what exactly it means that conscious mental states are “subjective” states. What exactly does it mean that conscious experience is often bound to an individual 1PP? We lack an empirically grounded theory of subjectivity, a model of the 1PP as a naturally evolved phenomenon. Having a 1PP is not a unitary, but a graded phenomenon, and research on mind wandering can make decisive contributions by functionally dissociating different levels.

^3^“Transparency” is a property of conscious representations, namely, that they are not experienced as representations. Therefore, the subject of experience has the feeling of being in direct and immediate contact with their content. Transparent conscious representations create the phenomenology of naïve realism. An opaque phenomenal representation is one that is experienced as a representation, for example in pseudo-hallucinations or lucid dreams. A transparent self-model creates the phenomenology of identification (section Mind Wandering as a Switch in the Unit of Identification). There exists a graded spectrum between transparency and opacity, determining the variable phenomenology of “mind-independence” or “realness.” Unconscious representations are neither transparent nor opaque. See Metzinger (2003b) for a concise introduction.

^4^This is not say that we never purposefully engage in daydreams or that there are never situations in which we are mind wandering while being passively aware of this fact. This is only to say that intentional episodes of daydreaming, to the extent that they do involve the phenomenology of AA and CA, thereby do not count as episodes of mind wandering, which refer only to unintentional episodes of stimulus-independent thought. One advantage of the terminological solution proposed here is exactly that it enables a continuous description of real-word cases: As long as the EAM still represents the ability to become an active attentional or cognitive agent, we have M-autonomy. What has been termed “zoning out” (unaware mind wandering) and “tuning out” (mind wandering with awareness) in the empirical literature (Smallwood et al., 2007 p. 524, 2008; Schooler et al., 2011, p. 323) can be nicely captured by this conceptual distinction.

^5^In adopting this terminological convention, I follow Marcel Brass [Brass and Haggard (2007)]; an excellent and helpful recent review is Filevich et al. (2012).

^6^See Brass and Haggard (2007); Campbell-Meiklejohn et al. (2008); Kühn et al. (2009). A helpful recent review of negative motor effects following direct cortical stimulation, listing the main sites of arrest responses and offering interesting discussion is Filevich et al. (2012)

^7^This passage draws on Metzinger (2013a). See also Filevich et al. (2012, 2013).

^8^Note, however, that there may also be methodological limits to how fine-grained such a distinction can be: in laboratory research on lucid dreams, dreams are typically scored as lucid if the dreamer submits predetermined eye movement signals while dreaming and later confirms having done so throughout awakening. At the same time, it is well known that lucidity fluctuates, and indeed it is often described as a balancing act between returning to a non-lucid dream and waking up (cf. Windt and Metzinger, 2007). Asking subjects to signal at certain time-intervals may be a way to ensure that lucidity and dream control were preserved throughout the dreams [cf. Stumbrys et al. (2013)]; however, even this method cannot rule out the occurrence of lapses of lucidity during the intervals, and asking subjects to signal lucidity continuously throughout the episode would surely be too demanding and disruptive. A related problem is that while the type of veto-autonomy required for M-autonomy is an ability, it can only be operationalized by investigating its realization, namely actual acts of terminating an ongoing episode of mind wandering. This, again, would seem to place certain methodological limits on how fine-grained the criteria for individuating such episodes can be. The challenge, then, is to develop a conceptual framework that is fine-grained enough to capture moment-to-moment fluctuations, but at the same time clear enough to be operationalized and allow for the formulation of clear scoring criteria.

^9^Schizophrenic delusions of thought control and thought insertion may be cases in which this ability breaks down and in which the respective thoughts cannot, as it were, be smoothly integrated into the self-model; see Metzinger (2003a, 2004); Stephens and Graham (2000) for discussion.

^10^Functionally, breathing and heartbeat are obviously quite different with respect to the availability for voluntary control. Breathing can be deliberately manipulated (even terminated at will), though not indefinitely. Phenomenologically, spontaneous thought is different from heartbeat and breathing. Mind wandering episodes can be terminated as soon as we have “come to ourselves” in the sense of having regained M-autonomy, though, again functionally speaking, not indefinitely. As opposed to breathing, the phenomenology of heartbeat is also much more subtle, and harder to influence deliberately. Moreover, breath and heartbeat share the functional property of being available through an interoceptive receptor system. Therefore, they generate a sensory phenomenology of self-representation. By contrast, as the human brain is devoid of any self-directed sensory channels or receptor systems, the relevant subset of DMN-activity cannot be informationally accessed through any perception-like causal links. Consequntly, the phenomenology of cognition must necessarily be a non-sensory phenomenology—although it can of course be about possible sensory perceptions, motor simulations, fantasy worlds, etc.

^11^For details, see Metzinger, 2003a, 2006, section 4. The philosophical notion of a “phenomenal model of the intentionality relation” (PMIR) is directly related to the idea of dynamically integrating top-down control (e.g., by the fronto-parietal control network) with subpersonal, bottom-up components (e.g., a subset of activity in the default-mode network) by creating an internal model of the whole organism as currently being directed at an object component, for example, a well-ordered train of thought; see Smallwood et al. (2012). The PMIR would then be the conscious correlate of this process, the phenomenal experience of what was termed CA in the main text.

^12^This is not to categorically exclude the possibility that the transition from stimulus-constrained thought to mind wandering and daydreaming could be gradual; perception itself is subtly modulated by expectation, prediction and anticipation, see Clark (2013); Madary (2013), and relatedly, mind wandering will not be an all-or-nothing affair, but will allow for various degrees and fluctuations of control and external-stimulus correlation. It is a familiar fact that mind wandering episodes are often triggered by perceptual stimuli. But the opposite can also be true: One study found mental time travel to be associated with subtle postural adjustments, with participants leaning backwards when asked to imagine the past and leaning to the front when asked to imagine the future cf. Miles et al. (2010a,b).

^13^A conceptual alternative is to describe them as states characterized by the maximal unit of identification, i.e., states in which the subject of experience identifies with the most general phenomenal property available, namely, the global quality of awareness as such (see Box 1 and Metzinger, 2013b for discussion). One may speculate that there are distinct phenomenal state-classes corresponding to each of the two logical possibilities.
